# ABO blood types and SARS-CoV-2 infection assessed using seroprevalence data in a large population-based sample: the SAPRIS-SERO multi-cohort study

**DOI:** 10.1038/s41598-023-30714-9

**Published:** 2023-03-23

**Authors:** Mélanie Deschasaux-Tanguy, Fabien Szabo de Edelenyi, Nathalie Druesne-Pecollo, Younes Esseddik, Julien Allègre, Bernard Srour, Pilar Galan, Serge Hercberg, Gianluca Severi, Marie Zins, Emmanuel Wiernik, SAPRIS-SERO study group, Fabrice Carrat, Pierre-Yves Ancel, Nathalie Bajos, Marie-Aline Charles, Gianluca Severi, Mathilde Touvier, Marie Zins, Sofiane Kab, Adeline Renuy, Stéphane Le Got, Céline Ribet, Mireille Pellicer, Emmanuel Wiernik, Marcel Goldberg, Marie Zins, Fanny Artaud, Pascale Gerbouin-Rérolle, Mélody Enguix, Camille Laplanche, Roselyn Gomes-Rimav, Lyan Hoang, Emmanuelle Correia, Alpha Amadou Barry, Nadège Senina, Gianluca Severi, Fabien Szabo de Edelenyi, Julien Allègre, Nathalie Druesne-Pecollo, Younes Esseddik, Serge Hercberg, Mathilde Touvier, Marie-Aline Charles, Pierre-Yves Ancel, Valérie Benhammou, Anass Ritmi, Laetitia Marchand, Cecile Zaros, Elodie Lordmi, Adriana Candea, Sophie de Visme, Thierry Simeon, Xavier Thierry, Bertrand Geay, Marie-Noelle Dufourg, Karen Milcent, Clovis Lusivika-Nzinga, Gregory Pannetier, Nathanael Lapidus, Isabelle Goderel, Céline Dorival, Jérôme Nicol, Olivier Robineau, Fabrice Carrat, Cindy Lai, Liza Belhadji, Hélène Esperou, Sandrine Couffin-Cadiergues, Jean-Marie Gagliolo, Hélène Blanché, Jean-Marc Sébaoun, Jean-Christophe Beaudoin, Laetitia Gressin, Valérie Morel, Ouissam Ouili, Jean-François Deleuze, Stéphane Priet, Paola Mariela Saba Villarroel, Toscane Fourié, Souand Mohamed Ali, Abdenour Amroun, Morgan Seston, Nazli Ayhan, Boris Pastorino, Xavier de Lamballerie, Xavier de Lamballerie, Fabrice Carrat, Mathilde Touvier

**Affiliations:** 1 Inserm U1153, Inrae U1125, Cnam, Nutritional Epidemiology Research Team (EREN), Epidemiology and Statistics Research Center – Université Paris Cité (CRESS), Sorbonne Paris Nord University, Bobigny, France; 2 UVSQ, Inserm, Gustave Roussy, “Exposome and Heredity” team, CESP UMR1018, Paris-Saclay University, Villejuif, France; 3 Department of Statistics, Computer Science and Applications “G. Parenti”, University of Florence, Florence, Italy; 4 Population-based Cohorts Unit, INSERM, UMS 011, Paris Saclay University, Université de Versailles Saint-Quentin-en-Yvelines, Université Paris Cité, Paris, France; 5 Unité des Virus Emergents (UVE), IRD 190, INSERM 1207, Aix Marseille Univ, Marseille, France; 6 Inserm, Institut Pierre-Louis d’Epidémiologie et de Santé Publique, Sorbonne Université, Paris, France; 7 Département de Santé Publique, APHP, Sorbonne Université, Paris, France; 8 Obstetrical, Perinatal and Pediatric Research Team (EPOPé), Centre for Research in Epidemiology and StatisticS (CRESS), Inserm, INRAE, Université de Paris, Paris, France; 9 Centre for Research in Epidemiology and StatisticS (CRESS), Inserm, INRAE, Université de Paris, Paris, France; 10 IRIS, UMR CNRS 8156, EHESS, Inserm U997, Aubervilliers, France; 11 EPIPAGE-2 Joint Unit, Paris, France; 12 ELFE Joint Unit, Paris, France; 13 Inserm, Paris, France; 14 Aviesan, Inserm, Paris, France; 15 Fondation Jean Dausset-CEPH (Centre d’Etude du Polymorphisme Humain), CEPH-Biobank, Paris, France

**Keywords:** Viral infection, Risk factors, Epidemiology

## Abstract

ABO blood type has been reported as a potential factor influencing SARS-CoV-2 infection, but so far mostly in studies that involved small samples, selected population and/or used PCR test results. In contrast our study aimed to assess the association between ABO blood types and SARS-CoV-2 infection using seroprevalence data (independent of whether or not individuals had symptoms or sought for testing) in a large population-based sample. Our study included 67,340 French participants to the SAPRIS-SERO multi-cohort project. Anti-SARS-CoV-2 antibodies were detected using ELISA (targeting the proteins spike (S) and nucleocapsid (NP)) and seroneutralisation (SN) tests on dried blood spots collected in May–November 2020. Non-O individuals (and especially types A and AB) were more likely to bear anti SARS-CoV-2 antibodies (ELISA-S, 2964 positive cases: OR_non-Ovs.O_ = 1.09[1.01–1.17], OR_Avs.O_ = 1.08[1.00–1.17]; ELISA-S/ELISA-NP/SN, 678 triple positive cases: OR_non-Ovs.O_ = 1.19 [1.02–1.39], OR_Avs.O_ = 1.19[1.01–1.41], OR_ABvs.O_ = 1.43[1.01–2.03]). Hence, our results provided additional insights into the dynamic of SARS-CoV-2 infection, highlighting a higher susceptibility of infection for individuals of blood types A and AB and a lesser risk for blood type O.

## Introduction

Since 2020, the world has been struggling with the COVID-19 (Coronavirus Disease 2019) pandemic caused by the SARS-CoV-2 (Severe Acute Respiratory Syndrome Coronavirus 2). The research community has mobilized to identify potential risk factors associated with SARS-CoV-2 infection and COVID-19 severity, in an effort to better understand the dynamic of infection, pinpoint individuals at higher risk and help prevent the disease and related adverse outcomes. Early reports comparing the distribution of ABO blood types between individuals infected or not by the SARS-CoV-2, suggested that ABO blood types could be related to the risk of SARS-CoV-2 infection^1–4^, as previously observed for SARS-CoV^5^ but also for other infectious diseases^6,7^. These studies and others published since then^8–11^ suggested that individuals with blood type O had a lower risk of SARS-CoV-2 infection and that type A individuals would be more at risk, with yet some remaining inconsistencies, especially for B or AB types for which results are less robust. However, these results were obtained in selected populations (e.g., patients, blood donors) of various sample size (mostly under 15,000) and mostly using PCR test results (e.g., from questionnaires or registries) hence reflecting a population that sought for testing, with the exception of a recent report using seroprevalence data in ≈32,600 blood donors^11^. In contrast, this short report provides insights into the associations between ABO blood types and SARS-CoV-2 infection assessed from seroprevalence data obtained between May and November 2020 through the systematic screening for anti-SARS-CoV-2 antibodies in a large population-based sample of French adults.

## Methods

The SAPRIS (“SAnté, Perception, pratiques, Relations et Inégalités Sociales en population générale pendant la crise COVID-19”) study was set up in April 2020 to investigate various aspects of the COVID-19 crisis (COVID-19 infection/diagnosis and experience of lockdown), based on a consortium of existing French prospective cohort studies. In May 2020, participants from the Constances, E3N-E4N and NutriNet-Santé cohort studies answering SAPRIS questionnaires were also invited to take part in the SAPRIS-SERO project which aimed to estimate the seroprevalence of antibodies against SARS-CoV-2 at the population level, as previously described^12,13^. Briefly, between May and November 2020 (during or at the end of the first wave, pre-vaccine period), participants received self-sampling kits by mail to collect dried blood spots from which were detected anti-SARS-CoV-2 antibodies (IgG) directed against the S1 domain of the spike protein (S) and the nucleocapsid protein (NP) using ELISA (Euroimmun®, Lübeck, Germany), as well as neutralizing anti-SARS-CoV-2 antibodies (SN) using an in-house micro-neutralization assay. ELISA-S and ELISA-NP tests were considered to be positive with an optical density ratio ≥ 1.1, indeterminate between 0.8 and 1.1, and negative < 0.8. The SN test was considered to be positive with a titre ≥ 40. ELISA-NP and SN tests were performed whenever the optical density ratio for ELISA-S was ≥ 0.7. Positivity (vs. negativity) to ELISA-S test and positivity (vs. negativity) to all three ELISA-S, ELISA-NP and SN tests (associated to more symptomatic COVID-19 in a previous study in SAPRIS-SERO^13^) were considered as outcomes. Characteristics of the participants including ABO and RhD blood types were collected from questionnaires as part of each cohort follow-up or from the shared SAPRIS questionnaire. Associations between the seroprevalence of antibodies against SARS-CoV-2 and blood types were studied using multi-adjusted logistic regression models stratified by cohort and including the following covariates: sex (men/women), age (≥ 18– < 40, 40–49, 50–59, 60–69, ≥ 70 years old), month of blood collection, residential area during the lockdown: city size (rural, < 20,000, 20–100,000, ≥ 100,000 inhabitants) and French administrative region. Additional covariates linked to the risk of SARS-CoV-2 infection were included in sensitivity analyses: weight status, smoking status, educational level, professional activity during lockdown, socio-professional category, number of individuals at home during lockdown and alcohol intake. All tests were two-sided and *P* < 0.05 was considered statistically significant. Analyses were carried out using SAS 9.4 (SAS Institute Inc., USA).

### Ethics approval and informed consent

The SAPRIS-SERO study (registered #NCT04392388) was conducted in accordance with the relevant guidelines and regulations and was approved by the ethics committee CPP Sud-Méditerranée III on April 27th 2020 and by the CNIL #920,193. All participants provided written or electronic informed consent for participation in each cohort and a specific electronic informed consent for participation in the SAPRIS-SERO study.

## Results

Our study included 67,340 participants, among which 2964 with a positive seroprevalence of anti SARS-CoV-2 antibodies according to the ELISA-S test and 64,376 participants who tested negative (respectively 678 triple positive and 64,011 triple negative to ELISA-S, ELISA-NP and SN tests). Characteristics of participants are shown in Table 1.

**Table 1:** Characteristics of the population, SAPRIS-SERO multi-cohort study, 2020.

	ELISA-S negative (N = 64,376)		ELISA-S positive (N = 2,964)		*P*
	N	%	N	%	
Gender					< 0.001
Male	21,174	(32.9)	832	(28.1)	
Female	43,202	(67.1)	2132	(71.9)	
Age (years)					< 0.001
≥ 18– < 40	6983	(10.8)	652	(22.0)	
40–49	10,340	(16.1)	988	(33.3)	
50–59	11,222	(17.4)	505	(17.0)	
60–69	13,224	(20.5)	334	(11.3)	
≥ 70	22,607	(35.1)	485	(16.4)	
Weight status^a^					0.006
Underweight	1992	(3.1)	110	(3.7)	
Normal weight	37,189	(57.8)	1749	(59.0)	
Overweight	17,763	(27.6)	745	(25.1)	
Obesity	5917	(9.2)	273	(9.2)	
Smoking status^a^					< 0.001
Non smoker	29,586	(46.0)	1605	(54.1)	
Smoker	5958	(9.3)	237	(8.0)	
Ex-smoker	26,351	(40.9)	997	(33.6)	
Educational level^a^					< 0.001
< High school	7262	(11.3)	171	(5.8)	
High school—undergraduate	28,063	(43.6)	1188	(40.1)	
Graduate or doctorate	24,074	(37.4)	1307	(44.1)	
Month of blood collection					< .0001
May	8004	(12.4)	663	(22.4)	
June	427	(0.7)	25	(0.7)	
July	38,143	(59.3)	1556	(52.5)	
August	13,120	(20.4)	540	(18.2)	
September	4607	(7.2)	178	(6.0)	
October	72	(0.1)	1	(0.0)	
November	3	(0.0)	1	(0.0)	
Residential area during the lockdown : city size^a^					< 0.001
Rural area	14,876	(23.1)	555	(18.7)	
< 20,000 inhabitants	10,923	(17.0)	441	(14.9)	
20–100,000 inhabitants	10,600	(16.5)	508	(17.1)	
≥ 100,000 inhabitants	27,230	(42.3)	1441	(48.6)	
Residential area during the lockdown : French administrative region^a^					< .0001
Haut de France	4116	(6.4)	179	(6.0)	
Auvergne- Rhône-Alpes	8316	(12.9)	351	(11.8)	
Occitanie	6979	(10.9)	188	(6.3)	
PACA	4306	(6.7)	133	(4.5)	
Corse	77	(0.1)	3	(0.1)	
Normandie	2463	(3.8)	87	(2.9)	
Ile-de-France	10,492	(16.3)	818	(27.6)	
Grand-Est	4679	(7.3)	389	(13.1)	
Bretagne	5066	(7.9)	143	(4.8)	
Pays de la Loire	3952	(6.1)	144	(4.9)	
Centre-Val-de-Loire	4224	(6.6)	174	(5.9)	
Bourgogne	2463	(3.8)	111	(3.7)	
Nouvelle Aquitaine	7228	(11.2)	243	(8.2)	
DOM-TOM	8	(0.0)	1	(0.0)	

^a^Missing values: N = 1602 (2.4%) for weight status, N = 2606 (3.9%) for smoking status, N = 5275 (7.8%) for educational level, N = 766 (1.1%) for city size and N = 7 (0.01%) for French administrative region.

Higher odds of presenting anti SARS-CoV-2 antibodies (Table 2) were observed in participants belonging to non-O blood types (ELISA-S: OR _non-O vs. O_ = 1.09 [1.01–1.17], ELISA-S/ELISA-NP/SN: OR _non-O vs. O_ = 1.19 [1.02–1.39]) and especially to blood type A (ELISA-S: OR_A vs. O_ = 1.08 [1.00–1.17], ELISA-S/ELISA-NP/SN: OR _A vs. O_ = 1.19 [1.01–1.41]) and AB (ELISA-S/ELISA-NP/SN: OR _AB vs. O_ = 1.43 [1.01–2.03]). In analyses combining ABO and RhD blood types, higher odds were observed in participants with blood types A- (ELISA-S: OR _A- vs. O+_  = 1.16 [1.00–1.35]), A + (ELISA-S/ELISA-NP/SN: OR _A+ vs. O+_  = 1.24 [1.03–1.48]) and AB + (which represented the majority of AB participants, ELISA-S: OR _AB+ vs. O+_  = 1.24 [1.02–1.51], ELISA-S/ELISA-NP/SN: OR _AB+ vs. O+_  = 1.60 [1.11–2.30]). No association was observed comparing RhD positive and negative blood types overall.

**Table 2:** Associations between ABO and RhD blood types and the seroprevalence of anti-SARS-CoV-2 antibodies, SAPRIS-SERO multi-cohort study, 2020.

	N (%)	ELISA-S				ELISA-S, ELISA-NP, SN		
		N positive/negative	OR [95%CI]^a^	P		N positive/negative^b^	OR [95%CI]	P
ABO				0.16				0.08
O	28,752 (42.7)	1216/27,536	Ref		27,648 (42.7)	260/27,388	Ref	
A	28,742 (44.4)	1342/28,574	1.08 [1.00–1.17]		28,742 (44.4)	323/28,419	1.19 [1.01–1.41]	
B	5,874 (8.7)	275/5599	1.10 [0.96–1.27]		5,613 (8.7)	58/5555	1.06 [0.80–1.42]	
AB	2,798 (4.2)	131/2667	1.14 [0.95–1.38]		2,686 (4.2)	37/2649	1.43 [1.01–2.03]	
Non-O	38,588 (57.3)	1748/36,840	1.09 [1.01–1.17]	0.03	37,041 (57.3)	418/36,623	1.19 [1.02–1.39]	0.03
ABO/RhD				0.15				0.05
O+	23,703 (35.2)	995/22,708	Ref		22,804 (35.3)	217/22,587	Ref	
O−	5,049 (7.5)	221/4828	1.03 [0.89–1.20]		4,844 (7.5)	43/4801	0.96 [0.69–1.33]	
A+	25,315 (37.6)	1120/24,195	1.07 [0.98–1.17]		24,344 (37.6)	286/24,058	1.24 [1.03–1.48]	
A−	4,601 (6.8)	222/4379	1.16 [1.00–1.35]		4,398 (6.8)	37/4361	0.91 [0.64–1.29]	
B+	4,886 (7.3)	224/4662	1.09 [0.94–1.27]		4,675 (7.2)	48/4627	1.04 [0.76–1.43]	
B−	988 (1.5)	51/937	1.21 [0.90–1.63]		938 (1.5)	10/928	1.14 [0.60–2.17]	
AB+	2,363 (3.5)	118/2245	1.24 [1.02–1.51]		2,266 (3.5)	35/2231	1.60 [1.11–2.30]	
AB−	435 (0.6)	13/422	0.70 [0.40–1.22]		420 (0.6)	2/418	0.48 [0.12–1.93]	
RhD				0.48				0.08
+	56,267 (83.6)	2457/53,810	Ref		54,089 (83.6)	586/53,503	Ref	
−	11,073 (16.4)	507/10,566	0.97 [0.87–1.07]		10,600 (16.4)	92/10,508	1.22 [0.98–1.52]	

^a^OR and 95%CI obtained from a multi-adjusted logistic regression model stratified by cohort and including the following covariates: sex (men/women), age (< 40ans, 40–49, 50–59, 60–69, ≥ 70 years old), month of blood collection, residential area during the lockdown: city size (rural, < 20,000, 20–10,000, ≥ 100,000 inhabitants) and French administrative region.

^b^A participant was considered positive (respectively negative) if positive (respectively negative) to all three ELISA-S, ELISA-NP, and SN tests. Among the N = 2964 individuals with a positive seroprevalence of anti SARS Cov-2 antibodies according to the ELISA-S test, N = 1185 were also positive according to the ELISA-NP test and 678 were positive according to all three ELISA-S, ELISA-NP, and SN tests.

Additional adjustments in sensitivity analyses did not change the results of increased odds of positive seroprevalence in non-O participants (ELISA-S: OR _non-O vs. O_ = 1.09 [1.01–1.18], ELISA-S/ELISA-NP/SN: OR _non-O vs. O_ = 1.19 [1.02–1.39]).

## Discussion

In this study, we used seroprevalence data obtained from screening a large population-based sample from 3 French prospective cohorts to assess the associations between ABO blood types and the risk of SARS-CoV-2 infection. Our results showed a decreased seroprevalence of anti-SARS-CoV-2 antibodies in type O individuals compared to all others. Among non-O blood types, types A and AB were more likely positive for SARS-CoV-2 infection. No association was observed with type B or RhD types.

These results confirmed prior observations obtained in other population settings, namely a decrease risk of infection for type O individuals and an increase risk for type A individuals, with some indication of increased risk for type AB individuals as well, thus strengthening the evidence towards a differential susceptibility of SARS-CoV-2 infection according to ABO blood types. Yet, most studies so far involved registry or hospital data of SARS-CoV-2 positivity from PCR test results in patients or blood donors in comparison to the general population^8–10^ and fewer studies used seroprevalence data, mostly in small samples, with the exception of a recent report in 32,600 blood donors^11^. In turn, our study used seroprevalence data and a screening design whereby a large population-based sample of 67,340 participants involved in the SAPRIS-SERO project received a dried blood spot sampling kit. This allowed us to test for SARS-CoV-2 infection in a single general population, regardless whether the participants sought testing or had symptoms, allowing detecting a large panel of infected cases.

This differential susceptibility to SARS-CoV-2 infection according to blood types could be due to the incorporation of ABH antigens on the envelop of SARS-CoV-2 virions, leading to a likelihood of infection from one individual to another following ABO incompatibility^9,10^. As type O individuals carry both anti-A and anti-B antibodies, they would be less susceptible to primary infection by SARS-CoV-2 viruses enveloped with type A or B-like patterns such as those excreted by non-O individuals. In contrast, type A individuals, carrying anti-B antibodies, would be susceptible to infection by viruses excreted by type O and type A individuals (i.e., 42% and 44% of the French population respectively), while type AB individuals carrying neither anti-A or anti-B antibodies would be susceptible to infection by individuals of all group types, which is why type A or AB are particularly at risk for SARS-CoV-2 infection. Type B individuals carrying anti-A antibodies are susceptible to infection by viruses excreted by type O and type B individuals but type B is less represented in the French population (10%) which may explain why no significant association was observed for these individuals. Another hypothesis, yet less supported by available data relates to the entry of SARS-CoV-2 in host’s cells, that would be facilitated by antigen A^9,14^. Finally, non-O individuals are at higher risk of thromboembolism, a common complication of COVID-19, hence beyond infection, non-O individuals could also be at higher risk of severe cases of COVID-19^9,10^.

Our study strengths pertained to the systematic screening for anti-SARS-CoV-2 antibodies targeting a large well-characterized sample, hence providing data from positive and negative cases within the same population, as well as the use of seroprevalence allowing the detection of cases that may or may not have symptoms or sought testing. Yet, some limitations should also be acknowledged. First, although large, our sample size may have still been limited to have sufficient power in analyses combining ABO and RhD blood types, especially for rarer blood types. Next, misclassification of individuals as ‘negative’ may have arisen from imperfect sensitivity of the ELISA-S test (85–90%, the specificity being > 95%)^15,16^ and from the decrease of anti-SARS-CoV-2 antibodies over time (with no detectable antibodies 4–5 months post-infection in half of participants in a recent report^11^). Yet, the sample collection was performed between May and November 2020, the first and second waves of the pandemic occurring in spring and fall 2020 in France, so that antibodies should still be detectable in most participants. Finally, the observational nature of the study makes it difficult to assess the extent to which participants have actually been exposed to the virus. Although lifestyle-related exposure to the virus is mostly unlikely to have been differential according to blood types, we adjusted our models on several covariates related to the spatial distribution of the epidemic since it could also relate to blood types, considering the distribution of ABO blood types in different regions or populations. However, we did not have access to racial and ethnic data as the collection of such data is generally not permitted in the framework of French cohort studies. Finally, our data only reflect infections occurring before November 2020, that is, before the emergence of variants but also before the large-scale vaccination campaigns.

To conclude, this study adds to the current body of evidence providing data on the association between ABO blood types and the risk of SARS-CoV-2 infection from screening a large sample from the general population using seroprevalence data. Our results confirmed a higher susceptibility of infection for individuals of blood types A and AB and a lesser risk for blood type O, thereby providing additional insights into the dynamic of SARS-CoV-2 infection. However, considering the magnitude of association and the current mechanistic hypotheses related to blood type incompatibility (hence a collective rather than individual type of protection), type O individuals are not without risk of infection and protective measures remain the best way of preventing SARS-CoV-2 transmission and infection.

## Data Availability

Data from the study are protected under the protection of health data regulation set by the French National Commission on Informatics and Liberty (Commission Nationale de l’Informatique et des Libertés, CNIL). The data can be made available upon reasonable request to the corresponding author (m.deschasaux@eren.smbh.univ-paris13.fr), after a consultation with the steering committee of the SAPRIS-SERO study. The French law forbids us to provide free access to SAPRIS-SERO data; access could however be given by the steering committee after legal verification of the use of the data. Please, feel free to come back to us should you have any additional question.
